# Arbuscular mycorrhiza enhance the rate of litter decomposition while inhibiting soil microbial community development

**DOI:** 10.1038/srep42184

**Published:** 2017-02-08

**Authors:** Heng Gui, Kevin Hyde, Jianchu Xu, Peter Mortimer

**Affiliations:** 1 Key laboratory for Plant Diversity and Biogeography of East Asia, Kunming Institute of Botany, Chinese Academy of Sciences, Kunming 650201, China; 2 World Agroforestry Centre, East and Central Asia, Kunming 650201, China; 3 Centre of Excellence in Fungal Research, Mae Fah Luang University, Chiang Rai 57100, Thailand; 4 School of Science, Mae Fah Luang University, Chiang Rai 57100, Thailand

## Abstract

Although there is a growing amount of evidence that arbuscular mycorrhizal fungi (AMF) influence the decomposition process, the extent of their involvement remains unclear. Therefore, given this knowledge gap, our aim was to test how AMF influence the soil decomposer communities. Dual compartment microcosms, where AMF (*Glomus mosseae*) were either allowed access (AM+) to or excluded (AM−) from forest soil compartments containing litterbags (leaf litter from *Calophyllum polyanthum*) were used. The experiment ran for six months, with destructive harvests at 0, 90, 120, 150, and 180 days. For each harvest we measured AMF colonization, soil nutrients, litter mass loss, and microbial biomass (using phospholipid fatty acid analysis (PLFA)). AMF significantly enhanced litter decomposition in the first 5 months, whilst delaying the development of total microbial biomass (represented by total PLFA) from T_150_ to T_180_. A significant decline in soil available N was observed through the course of the experiment for both treatments. This study shows that AMF have the capacity to interact with soil microbial communities and inhibit the development of fungal and bacterial groups in the soil at the later stage of the litter decomposition (180 days), whilst enhancing the rates of decomposition.

Litter decomposition refers to several physical, chemical and biological processes that convert plant litter to simple chemical compounds, such as carbon dioxide, water and inorganic ions, which can be then absorbed by plant roots and other soil organisms^1^. Litter decomposition is a key process for terrestrial ecosystems, forming a critical component of the nutrient and carbon cycles^2^. The rate of litter decomposition is controlled by several abiotic and biotic factors including temperature, soil moisture, substrate quality and diversity soil organisms^3^,^4^.

Mycorrhizal fungi are an important group of organisms involved in litter decomposition, within this group of organisms arbuscular mycorrhizal fungi (AMF) comprise the largest component, forming symbiotic associations with the roots of about 80% of all plant species^5^. Amongst a wide array of benefits, AMF are known to improve host nutrient uptake (primarily nitrogen (N) and phosphate (P))^6^,^7^,^8^,^9^. However, AMF are not known to have saprotrophic capabilities, and, as of yet, have not been shown to directly degrade organic matter^10^, although it has been shown that AMF can influence the decomposition process^11^,^12^,^13^. A good example of AMF influence on decomposition is provided by Hodge *et al*.^14^, who reported that the presence of AMF significantly enhanced the host plant’s N acquisition from a litter patch and resulted in an increased C loss from the litter patch. However, the mechanisms by which AMF are able to influence litter decomposition remains unclear. As the soil microbial community largely mediates the decomposition process, it is possible that AMF can exert an indirect influence on this process through regulating free-living groups of decomposers in the soil^14^. Nuccio *et al*.^15^ reported that during litter decomposition the presence of AMF altered approximately 10% of the bacterial community. Toljander *et al*.^16^ investigated the effect of AMF hyphal exudates on a soil bacterial community and reported an increase in bacterial growth as well as significant changes in the bacterial community composition. Leifheit *et al*.^17^ reported that AMF inoculation decreased the decomposition rates of woody material, with the presence or absence of plant roots having no effect on the results. These reports highlight the potential roles of AMF in the decomposition process, yet there is still little evidence confirming the exact role of AMF in mediating the decomposition of organic material in the soil.

Past studies have provided crucial insights into the mechanisms underlying the influence of AMF on soil decomposer communities, and further provided the tools and models with which to study these interactions^12^,^18^,^19^. However, these studies have not investigated the influence of AMF on indigenous leaf litter in natural soils, but rather focused on model plants in experimental soil mediums. Thus we set out to test the influence of AMF on the decomposer communities found in soil from a subtropical forest soil in southwestern China using leaf litter from indigenous plant species. Specifically, our aims were to investigate the impact of AMF on the decomposition rate of leaf litter in soils taken from a natural forest; and to determine how the microbial communities in these soils respond to the presence of AMF. Our hypothesis being that AMF will enhance the rate of litter decomposition through increasing the activity levels of associated soil bacterial and fungal communities.

## Results

### AMF root colonization

In the AM+ treatment, the root length colonized by AMF was initially 24.9% at T_90_ and continued to increase to 72.8% in the T_180_. No significant levels of AMF colonization were detected in AM− treatment (Supplemental Figure S1).

### Litter decomposition

**Figure 1:**
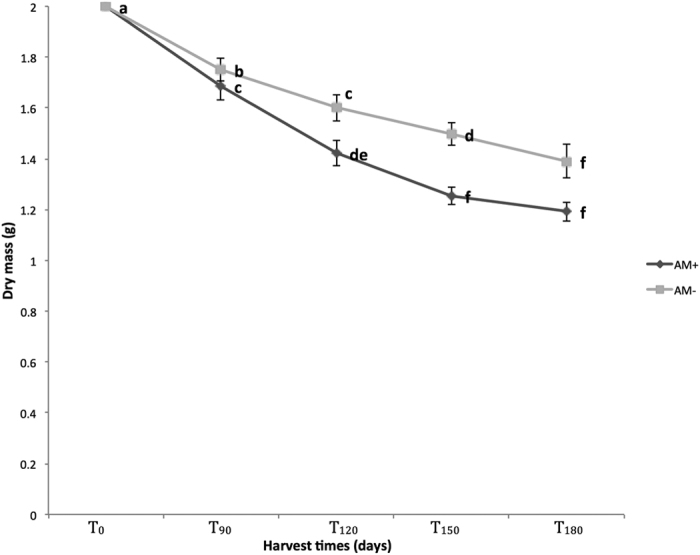
Dry mass loss of *Calophyllum polyanthum* leaf litter under two treatments (AM+ and AM−), at different sampling times. AM+ represents the treatment containing arbuscular mycorrhizal fungi and AM− represents the mycorrhizal free treatment. Different letters indicate significant differences (*p* < 0.05, n = 4, ± SE).

For both the AM− and AM+ treatments litter dry mass was reduced significantly over time. At the T_180_ harvest a 30.5% and 40.5% dry mass loss was recorded for AM− and AM+ treatments respectively. At T_90_, T_120_ and T_150_, the AM+ treatment had a significantly greater mass loss than AM− treatment. Furthermore, in the AM−treatment the dry mass loss significantly decreased for each sampling time, while in the AM+ treatment there were no differences in the dry mass loss between T_150_ and T_180_ (Fig. 1).

### Soil nutrients

No significant differences were recorded in the total carbon (TC), total N (TN), total P (TP), total potassium (TK) and the ratio of TC and TN (C: N) between treatments or over time (Table 1). However, for both treatments, available N (AN) declined over the duration of the experiment and was significantly lower at T_150_ and T_180_ compared to T_0_ and T_90_ (Table 1). AN did not differ significantly between the AM+ and AM− treatments. The available P (AP) found in the AM+ treatment was significantly lower by T_180_ than T_90_ (Table 1), additionally there was a noted peak in available P at T_150_ in the AM− treatment. There were no significant differences between the AM+ and AM− treatments during the experiment for AP (Table 1).

### Soil microbial communities

**Figure 2:**
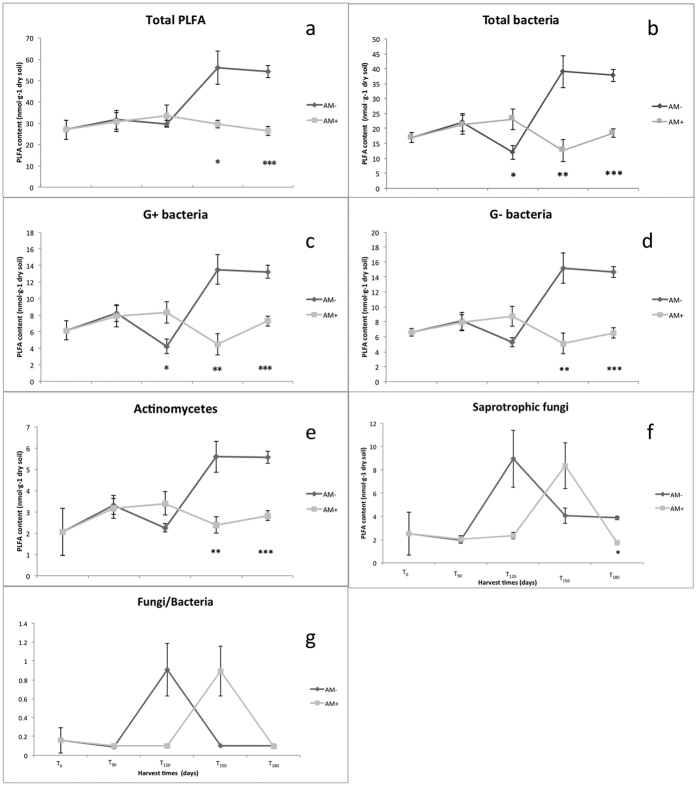
The change in soil phospholipid fatty acid analysis (PLFA) contents (nmol·g-1) for different sampling times. (**a**) Total PLFA, (**b**) Total bacteria, (**c**) Gram-positive bacteria, (**d**) Gram-negative bacteria, (**e**) Actinomycetes, (**f**) Saprotrophic fungi, (**g**) The ratio of saprotrophic fungi to bacteria. AM+ represents the treatment containing arbuscular mycorrhizal fungi and AM− represents the mycorrhizal free treatment. Mean (n = 4) values ± SE are shown; in each plot *indicates *p* < 0.05, **indicates *p* < 0.01, ***indicates *p* < 0.001.

Initially (T_0_-T_120_) no differences were observed in the total soil microbial biomass between treatments, however at T_150_ and T_180_ the biomass of the general microbial community of the AM− treatment had risen to levels significantly greater than those of the AM+ treatment, which remained unchanged over the course of the experiment (Fig. 2a). The soil bacterial biomass was significantly greater in the AM+ treatment at T_120_, however during the final two harvests (T_150_ and T_180_) this changed and the bacterial biomass was significantly greater in the AM− treatment (Fig. 2b). The average PLFA values for Gram−positive (G + ) and Gram−negative (G−) bacteria, as well as the actinomycetes all followed a similar trend in community dynamics as that of total bacteria (Fig. 2c,d and e). More detailed analysis of specific PLFA markers for G + and G- bacteria showed a similar trend as that of the average values for G+ and G- bacteria. AM fungal inoculation significantly decreased the following specific PLFA markers for G+ bacteria: i15:0, a15:0, i16:0, i17:0 and a17:0 at T_150_ and T_180_ (Supplementary Figure S2). Similarly, AM fungal inoculation significantly decreased the following specific PLFA markers for G− bacteria: 16:1 ω 7c, 18:1 ω 11c, cy17:0 and cy19:0 at T_150_ and T_180_ (Supplementary Figure S3). In comparison with the bacterial groups, the saprophytic soil fungi displayed a different response to the presence of AMF. The biomass of saprotrophic fungi in the AM− treatment increased nearly 3.5 fold between T_90_ and T_120_ (Fig. 2f). However, by T_150_ the fungal biomass of the AM− treatment had dropped by 55% compared with the biomass at T_120_, and declined a further 4.2% by T_180_ (Fig. 2f). A similar trend was observed in the AM+ treatment, except for a one-month lag compared to the AM− treatment, the fungal biomass increased by 3 fold from T_120_ to T_150_, however the biomass values at T_180_ dropped to a level similar to that of the biomass observed at T_0_ (Fig. 2f). By T_180_ the fungal biomass of the AM− treatment was significantly higher than that of the AM+ treatment (Fig. 2f).

The ratio of fungi to bacteria, reached its peak value of 0.91 at T_120_ for the AM− treatment, again, there was a one-month lag for the AM+ treatment, which only peaked at T_150_ with a value of 0.89 (Fig. 2g).

Additionally, at T_120_, Shannon’s and Simpson’s indices for the AM− treatment were significantly lower (*p* < 0.05) than the AM+ treatment. However, at T_150_, the Shannon’s and Simpson’s indices for the AM− treatment were significantly higher than the AM+ treatment (Table 2). For the Pielou evenness index, there were no significant differences among the treatments during litter decomposition (Table 2).

### Principal component analysis

**Figure 3:**
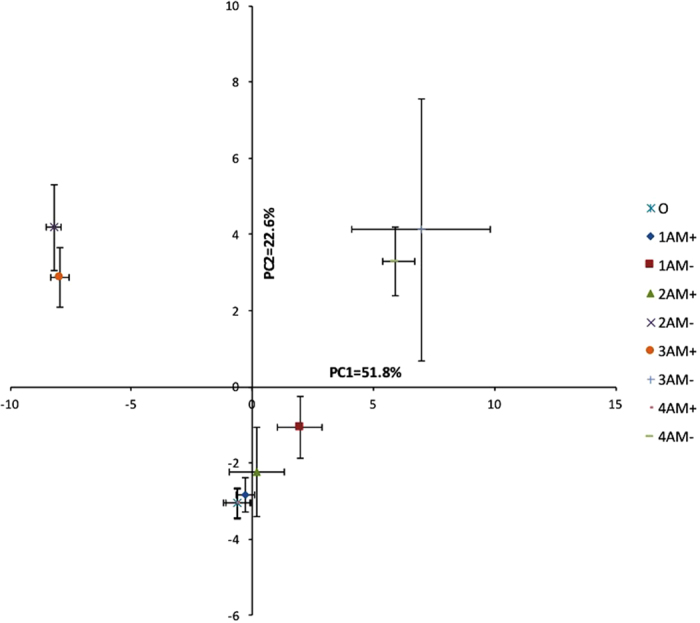
Principal component analysis of the soil microbial communities according to the phospholipid fatty acid analysis (PLFA) profile for different treatments and sampling times. Each symbol represents the mean value ( ± SE, n = 4) derived from soil samples taken at each harvest time. Symbol captions are described as follows: the first number (0, 1, 2, 3 and 4) represents the harvest times T_0_, T_90_, T_120_, T_150_ and T_180_ respectively. AM+ represents the treatment containing arbuscular mycorrhizal fungi and AM− represents the mycorrhizal free treatment.

PC1 and PC2 captured 51.8% and 22.6% of the total data variability. The principal component analysis (PCA) analysis also indicated that for each month, the soil microbial groups from the two treatments significantly separated along the two axes. Additionally, this separation increased with time. Furthermore, this analysis indicated that the soil microbial groups from the AM+ treatment differed significantly at T_150_ and T_180_ (Fig. 3).

### Redundancy analysis

**Figure 4:**
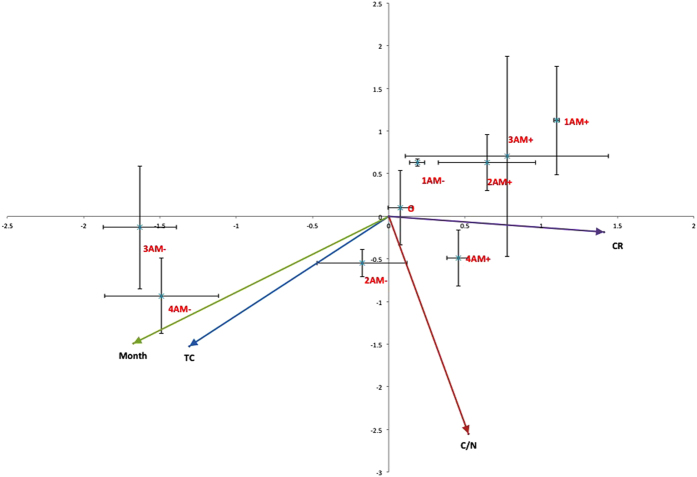
Redundancy analysis of the soil microbial community composition based on the phospholipid fatty acid analysis (PLFA) profiles from the different treatments (AM+ and AM−), at different sampling times. Each symbol represents the mean value ( ± SE, n = 4) derived from soil samples taken at each harvest time. Symbol captions are described as follows: the first number (0, 1, 2, 3 and 4) represents the harvest times T_0_, T_90_, T_120_, T_150_ and T_180_ respectively. AM+ represents the treatment containing arbuscular mycorrhizal fungi and AM− represents the mycorrhizal free treatment. Note: month (different sampling times), TC (total soil carbon), C/N (the ratio of total soil carbon and total soil nitrogen) and CR (arbuscular mycorrhizal fungal percentage colonization).

Redundancy analysis (RDA) results revealed that total carbon (TC) (*F* = 0.94, *p* = 0.036), harvesting time (month) (*F* = 1.56, *p* = 0.012), C/N (*F* = 2.23, *p* = 0.028), and percentage root colonization (CR) (*F* = 1.31, *p* = 0.024) were the 4 most dominant factors significantly correlated to the changes observed in the soil microbial communities (Fig. 4). TN (*F* = 4.99, *p* = 0.007) and TK (*F* = 0.96, *p* = 0.032) also significantly contributed to the variation in PLFA profile. All the environmental factors explained 93.0% of variance in axis 1 (Eigenvalue = 10.71) and 4.9% (Eigenvalue = 0.56) in axis 2. Harvesting time, TC and CR were highly correlated with axis 1, whereas C/N was highly correlated with axis 2 (Fig. 4).

## Discussion

The inoculation of *Trifolium repens* with *Glomus mosse* resulted in a faster litter decomposition process and a delay in the development of soil decomposer communities, suggesting that AMF directly influenced this process. These results partially disprove our hypothesis, although AMF did increase the rate of decomposition, this was not achieved by AMF enhancing the activities of the soil fungal and bacterial groups involved in the decomposition process. These findings are in agreement with past studies showing that AMF can indirectly increase litter decomposition rates^11^,^12^,^19^. However, this is amongst the first study to report that the increased rate of litter decomposition is attributed to AMF.

The AMF induced increase in litter decomposition, which was most apparent around T_120_ and T_150_, did not carry through to the end of the experiment, with no significant differences between the treatments by T_180_. These findings are in line with those of Herman *et al*.^12^ and Hodge *et al*.^14^ who observed a similar accelerating effect on litter decomposition due to AMF, however they noted these changes after a much shorter time period, after approximately 40–50 days. These two authors argued that AMF hyphae play a direct role in influencing the rate of litter decomposition, as no changes in the soil microbial communities were observed in their experiments, despite the fact that AMF are presumed to have no saprophytic capacity. However, it appears that despite the evidence of AMF positively influencing the rate of leaf litter decomposition, AMF does not have a positive influence on the decomposition of woody material. Leifheit *et al*.^17^ reported that AMF significantly inhibited the decomposition of woody material, over a five-month period. Therefore, the evidence provided by our study and the works of Herman *et al*.^12^ and Hodge *et al*.^14^, indicates that AMF enhance the decomposition rates of leaf litter, although the mechanisms by which this occurs remain unclear.

Time was shown to be one of the significant factors driving microbial community divergence, both within treatments and across the treatments. This is in accordance with past studies that reported different nutrient dynamics for the earlier and later stages of litter decomposition^12^,^17^,^20^. The temporal changes observed in our study include the changes in decomposition between treatments as well as changes in the microbial communities. All of which have been reported in the past, Herman *et al*.^12^ reported on the effect of AMF on decomposition as well as changes in the microbial communities. However, this is the first report showing that AMF suppress the development of the broader soil microbial community, and delay the development of both the saprophytic fungal and bacterial communities in the soil, whilst simultaneously enhancing the decomposition of litter.

Initially, from T_0_ to T_120_, there was no mycorrhizal effect on the broader soil community, however after this period, AM suppressed any potential development that this community might have undergone, as evidenced by the substantial growth that took place in the AM− treatment after T_120_. Furthermore, this suppression of the soil microbial community coincided with the highest levels of AM root colonization (above 65%), indicating that AM fungi were well established on the root systems and in the surrounding soils. In addition, percentage colonization was shown to be a significantly influencing factor in the microbial community development. It appears there was a critical level of mycorrhizal colonization required before AMF was established enough to influence the soil microbial groups, and the benefits of AMF on litter decomposition became apparent. Provided AMF colonization levels remain above that threshold (65% for our study) it is likely that the impact of AMF on the decomposers and litter decomposition would continue for some time after T_180_. Although to say exactly how long this influence would remain in place would be speculative at best, and most likely a factor of litter quality and composition. The observed suppression of the microbial communities in our study is in agreement with the work of Welc *et al*.^21^ and Mechri *et al*.^22^. However, when assessing the effects of AMF on the different microbial groups, different patterns emerged.

The saprophytic fungi in the AM+ treatment showed a one-month lag in development compared to the AM− treatment, and by the final harvest at T_180_ the levels of saprophytic fungal biomass were significantly lower in the AM+ treatment. Olsson *et al*.^23^ reported similar findings, showing the AM fungi can inhibit the development of the saprophytic fungi. However, this one-month lag in the development of the saprophytic fungal community in the AM+ treatment of our study appears to be a novel finding, for example, Herman *et al*.^12^ found that saprophytic fungi followed the same trend in development when comparing treatments with and without AM. Unlike the one-month delay in the development of the saprophytic fungal community, AM suppressed the bacterial community development to a point where the bacterial biomass after T_180_ was similar to at the start of the experiment, whereas the AM− treatment had significantly greater levels of bacterial biomass (total bacterial, G+, G−, and actinomycetes) by this stage. These findings are not novel, both Welc *et al*.^21^ and Mechri *et al*.^22^ reported that *G. intraradices* and *G. mossee* reduced the levels of bacteria in soils associated with different AM host plants.

When assessing community dynamics and the interactions between the respective groups in the soil, our results indicate that saprophytic fungi may inhibit the growth of the soil bacterial groups. At T_120_ the biomass of the saprophytic fungi in the AM− treatment peaked, whilst there was a simultaneous drop in the biomass of the respective bacterial groups. This was not observed for the AM+ treatment due to the inhibition of the saprophytic fungi by the AMF. This pattern of inhibition has been observed in other studies^24^,^25^, which indicated that saprophytic fungi dominate the early stage of decomposition, by suppressing the development of other microbial groups, such as bacteria. This pattern on development was also reflected in the fungi: bacteria ratio which followed a similar pattern to that of the fungal communities, providing further evidence of the influence of soil fungi over that of the bacterial groups. For both treatments, one month after the peak in the fungi: bacteria ratio there was a pronounced dip to levels similar to that at the start of the experiment, reflecting the diminishing role of soil fungi in the later stages of decomposition and the increasing role of bacteria. This succession within decomposer groups has been observed in a number of other studies^12^,^25^,^26^.

The observed changes within the decomposer communities of both treatments also impacted on the soil nutrient profiles within the forest soils used in our study. By the end of the experiment, the amount of AP was lower in the AM+ treatment, which would be consistent with current theory that AM are efficient at mining soil P and supplying this to the host plant^5^. However, this was the only notable difference between treatments, the remaining changes in nutrition were an outcome of time rather than treatment. AK was significantly lower by the end of the experiment for both treatments, similarly, AN was lower in both treatments from T_150_. One significant trend in nutrient dynamics was observed in how the trends associated with available P changed over time. In the AM+ treatment, there was a predictable and steady decline in the amount of available P, however, for the AM− treatment, the amount of available P increased between T_90_ and T_120_, before declining again at T_150_. This peak in P availability likely contributed towards the peak noted in the levels of saprophytic fungi at the same time period, supporting the activity of these fungi^27^. A subsequent drop in the biomass of saprophytic fungi was then observed at the same time that AP declined. However, this was the only change in soil nutrient status that affected the microbial communities dynamics, which were most strongly influenced by time and treatment (mycorrhizal colonization levels).

## Conclusion and Outlook

Our study provides clear evidence that AMF contributes towards the decomposition of organic matter, whilst simultaneously inhibiting the soil microbial community, most significantly during the later stages of litter decomposition. This interaction provides new insight into how AMF interact with soil microbial groups.

The interactions between AMF and soil microbial communities are only now beginning to be understood and it is clear there are direct interactions between these organisms, influencing the decomposition process. However, to fully appreciate and understand the extent of these interactions more detailed approaches to research are required, such as the use of next-generation sequencing, mRNA analyses, enzyme kinetics, and isotopic labeling experiments. These techniques will provide insight into which groups of organisms are being influenced (suppressed or enhanced activities) by AMF and how the nutrient cycling processes are affected by these interactions.

## Methods

### Mycorrhizal microcosm: design and set-up

Our experiments were conducted using an acrylic microcosm unit, which consisted of two compartments; the design was modified from that of Herman *et al*.^12^. The first compartment, used to pot the host plant, was filled with sterilized vermiculite and fine gravel (*ca.* 0.3 cm diameter), which was evenly mixed in a 1:1 ratio. For the AM+ treatment 20 g of AMF inoculum (*Glomus mosseae*) was added to the potting medium. Forest soil was placed in the second compartment and a litterbag (5 cm * 5 cm) buried in the soil at a depth of 5 cm. The litterbag was made of 200μm nylon mesh. The litter comprised dried leaves of *Calophyllum polyanthum* Wall. ex Choisy (Clusiaceae), an indigenous tree to Yunnan Province, and one of the dominant species from the forest used to collect the soil. The leaves were collected from nursery grown *C. polyanthum* saplings, and were oven dried at 65 °C to a constant weight. The dried leaves were cut into small pieces (*ca.* 5 mm * 5 mm), 2 g of which were put into the litterbags. The litter was cut into smaller units to ensure a larger surface area available for decomposition, thus ensuring a decomposition response for our experimental purposes. This was done according to the work of Kuramae *et al*.^26^. The two compartments (20 cm * 10 cm * 15 cm, length, width, depth, respectively) of the microcosm were separated by an air gap, which was drilled with evenly spaced holes (4 mm in diameter) and covered by 20 μm nylon mesh on both sides, the mesh allows AMF hyphae to pass through, but not plant roots (Supplemental Figure S4).

The microcosms were divided into two treatments (AM+ and AM−), based on the host plant being inoculated with AMF or not. Four replicates were set for each treatment. Additionally, 4 time-phase samplings were taken at monthly intervals. Thus, in total, 32 microcosms were set up for the experiment. All the microcosms were randomly placed in a greenhouse with daily temperature ranging from 20 to 25 °C. Plants received natural light only and no rainwater.

### Plant growth and AMF inoculation

The soil used in the microcosms was collected from a subtropical forest located in southwestern China (N 21°31′42.13″, E 100°29′41.87″). The top 5 cm of soil was collected after first removing the litter layer. The soil is classified as Torrents Vertisols according to the Soil Survey Staff^28^. The soil was sieved using a 2 mm mesh in order to remove any stones or root material. The soil was then placed into the relevant microcosm compartments and the AM treatment received inoculum at the same time. The Institute of Plant Nutrition and Resources, Beijing Academy of Agriculture and Forestry Sciences (Beijing, China) provided the AMF inoculum, *G. mossea*.

*Trifolium repens* L. cv. Milkanova (Fabaceae) was selected as the host plant and 0.2 g of surface sterilized seeds were planted in the first chamber of the microcosm (Supplemental Figure S4). The chamber housing the host plant received 10 ml of distilled water twice a week and 10 ml of modified Long Ashton nutrient solution once a week^29^. The chamber with the forest soil and litterbag received 10 ml of distilled water once a week in order to maintain the moisture levels. After two weeks the N and P concentrations in the Long Ashton solution was diluted to 1/10 of the original concentration (34 mg∙L^−1^ NaNO_3_ + 21.4 mg∙L^−1^ NH_4_Cl, 29.2 mg∙L^−1^ NaH_2_PO_4_∙2H_2_O + 4.7 mg∙L^−1^ Na_2_HPO_4_∙12H_2_O) according to Leigh *et al*.^30^ and adjusted to pH 7.0 with NaOH.

### Plant and soil sampling

The soil was collected from the forest (T_0_) as four subsamples which were bulked into one composite sample and then preserved at 4 °C for the later analysis. The first time of sampling from the microcosm was conducted 90 days (T_90_) after planting, subsequent sampling times were at 120 days (T_120_), 150 days (T_150_) and 180 days (T_180_) after planting. At each sampling event plants were harvested and soil samples taken. Soil samples included 20 g of the soil from around the litterbag, this was split into two subsamples, 10 g was preserved at 4 °C for later chemical analyses, and the remaining 10 g was immediately freeze-dried and then preserved at −80 °C for later phospholipid fatty acid analysis (PLFA). The litterbag was removed from the soil and the litter from the bag was then dried at 65 °C to a constant weight and weighed to determine mass loss. In order to test how the influence of AMF on litter decomposition varies with the time, the percentage of decomposition rate under AM fungal inoculation was calculated each sampling time.

### Soil chemical analyses

Soil organic matter was determined by Dumas combustion^31^, and total N usinga semi-micro Kjeldahl apparatus^32^. Total P and potassium (K) were measured spectro-photometrically after digesting a mixture of concentrated H_2_SO_4_ and H_2_O_2_^33^. Hydrolysable N was analyzed through a reaction with iron (II) sulfate and sodium hydroxide in a diffusion procedure^34^. Available P and K were determined using ammonium fluoride and ammonium acetate^35^. All the chemical analyses were conducted in Yunnan Agriculture Academy, Yunnan Province, China.

### Percentage mycorrhizal colonization

The percentage of *T. repens* root length colonized by AMF was determined using fresh root samples. Root tips (2 cm in length) were washed in distilled water and then rinsed with 10% KOH, which was stained with pen ink according to the methods of Vierheilig *et al*.^36^. The percentage colonization was calculated by a modified line intersection method^37^.

### Soil lipid extraction and PLFA analysis

Lipid extraction and PLFA analyses were performed in the laboratories of the South China Botanical Gardens, Chinese Academy of Sciences, using the modified Bligh and Dyer-method^38^,^39^. Briefly, a subsample (8 g) of the freeze-dried soil sample was extracted with a chloroform-methanol-citrate buffer mixture (1:2:0.8), and the phospholipids were separated from other lipids on a silicic acid column. The phospholipids were subjected to a mild alkaline methanolysis and the resulting fatty acid methyl esters (FAMEs) were analyzed using a gas chromatograph/mass spectrometry (GC-MS) system. The MIDI Sherlock^TM^ Microbial Identification System (MIDI, Newark, DET) identified all of the specific signatures derived from the GC-MS for each of the individual FAMEs.

Standard nomenclature for fatty acid characterization was used^40^. Individual fatty acids were designated according to convention by the total number of carbon atoms, number of double bonds, then followed by the position of the double bond from the methyl-end of the molecule. For unsaturated fatty acids, ωn follows, where n indicates the position of first carbon of the double bond from the aliphatic end of the molecule. The prefixes i and a indicate iso- and anteiso-branching, respectively, and cy indicates cyclopropane fatty acid. Me refers to the position of the methyl group from the carboxyl-end of the chain. Fatty acids were classed into different groups (bacterial, fungal and actinomycete), and used to indicate their respective biomass estimates, according to Leckie^41^.

On average, 42 fatty acids were detected for each soil sample. Only 31 of them were identified as a specific microbial group through the use of their biomarkers. Total microbial biomass (total PLFAs) was expressed as the sum of all the extracted PLFAs. PLFAs that correspond to carbon chain lengths of 12–20 carbons were generally associated with microorganisms^40^. Gram positive bacteria were represented by the PLFAs: i14:0, i15:0, a15:0, i16:0, i17:0, a17:0, and i18:0^42^,^43^, and Gram negative bacteria were represented by the PLFAs: 16:1 ω11c, 16:1 ω7c, 17:1 ω8c, 18:1 ω7c, cy17:0, cy19:0, and 15:0 3OH^40^,^44^. Other saturated straight-chain PLFAs (14:0, 15:0, 16:0, 17:0, 18:0) were identified as non-specific bacteria^45^. The PLFAs 18:1 ω9c, 18:2 ω6,9c, and 18:3 ω6,9,12c were used as the indicators of fungi^46^, and 16:1 ω5c in the soil was used as an indictor of AMF^40^. Finally, 10Me16:0 and 10Me18:0 represent the actinomycetes^31^. The ratio of fungal to bacterial PLFAs and Gram positive to negative bacterial PLFAs were used as indicators of changes in the relative abundance of these microbial groups^47^.

### Statistical analyses

The treatment effects on soil chemicals and microbial groups were determined by one-way analysis of variance (ANOVA) and student’s T-test. The ANOVA was run separately for each month between treatments. Before analysis, all the datasets were pre-tested to check the normality and equality of the variance to determine if they fulfilled the necessary assumptions for parametric testing, any datasets that failed to meet these criteria were tested using non-parametric tests (Mann-Whitney U test). Analysis for all data was carried out in SPSS version 20.0 (SPSS, Chicago, IL).

Variability within the PLFA profiles was determined using PCA based on the content of each of the detected fatty acids. RDA was performed to determine which environmental factors had the greatest influence on the soil microbial community composition. Before running the PCA and RDA analyses, detrended correspondence analysis (DCA) was used to calculate the gradient length of the datasets of PLFA markers, using CANOCO software (Microcomputer Power, Inc., Ithaca, NY). Gradient length was determined to be 1.506, which indicated that PCA and RDA were the appropriate liner models. Soil properties and other environmental factors were tested for significant contribution to the explanation of the variation in the PLFA data with the Monte Carlo permutation test (*p* < 0.05). All the PCA and RDA calculations were run using the vegan package in R studio (R core team, 2013). The Euclidean measure of distance in vegan package was used in this analysis to select the most discriminating environmental variables. Spearman’s correlation analysis was used in case of nonparametric data. All effects noted were significant at the *p* < 0.05 levels.

Soil microbial community diversity indices, based on the PLFA profiles, were calculated according to:


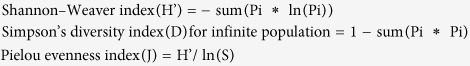


Where Pi refers to the ratio of the content of each fatty acid to the total content of one soil sample. Each fatty acid was considered as representative of one species^48^,^49^. S refers to the total number of detected PLFAs for each soil sample.

## Additional Information

**How to cite this article**: Gui, H. *et al*. Arbuscular mycorrhiza enhance the rate of litter decomposition while inhibiting soil microbial community development. *Sci. Rep.* **7**, 42184; doi: 10.1038/srep42184 (2017).

**Publisher's note:** Springer Nature remains neutral with regard to jurisdictional claims in published maps and institutional affiliations.

## Supplementary Material

Supplementary Information

## Figures and Tables

**Table 1:** Soil chemical properties for the different treatments (AM+ and AM−), at different sampling times (T_0_, T_90_, T_120_, T_150_ and T_180_).

Harvest time (days)	T_0_	T_90_		T_120_		T_150_		T_180_	
Soil property		AM+	AM−	AM+	AM−	AM+	AM−	AM+	AM−
TC (g/kg)	107.79 ± 1.42^a^	107.58 ± 2.34^a^	112.77 ± 2.54^a^	106.61 ± 1.85^a^	104.88 ± 2.47^a^	104.54 ± 1.2^a^	109.88 ± 2.23^a^	107.13 ± 2.09^a^	104.09 ± 0.79^a^
TN (g/kg)	4.81 ± 0.17^a^	4.72 ± 0.14^a^	4.81 ± 0.01^a^	4.7 ± 0.08^a^	4.61 ± 0.08^a^	4.73 ± 0.17^a^	5.24 ± 0.16^a^	4.59 ± 0.02^a^	4.82 ± 0.08^a^
TP (g/kg)	0.77 ± 0.01^a^	0.75 ± 0.01^a^	0.75 ± 0.01^a^	0.76 ± 0.02^a^	0.78 ± 0.01^a^	0.77 ± 0.01^a^	0.79 ± 0.01^a^	0.81 ± 0.03^a^	0.81 ± 0.01^a^
TK (g/kg)	4.68 ± 0.03^a^	4.68 ± 0.08^a^	4.71 ± 0.1^a^	4.58 ± 0.08^a^	4.65 ± 0.08^a^	4.56 ± 0.07^a^	4.64 ± 0.03^a^	4.64 ± 0.03^a^	4.83 ± 0.06^a^
AN (mg/kg)	397.15 ± 13.38^ab^	398.41 ± 11.75^ab^	424.12 ± 10.53^a^	385.56 ± 4.95^abc^	381.28 ± 6.33^abc^	352.72 ± 9.44^c^	364.14 ± 3.59^bc^	349.86 ± 8.2^c^	351.29 ± 3.69^c^
AP (mg/kg)	13.35 ± 1.02^ab^	13.07 ± 1.04^a^	13.81 ± 0.68^a^	12.92 ± 0.77^ab^	15.67 ± 0.62^a^	10.35 ± 1.11^ab^	9.82 ± 2.63^ab^	7.8 ± 0.98^b^	12.31 ± 1.28^ab^
AK (mg/kg)	68.13 ± 5.98^b^	80.63 ± 2.37^ab^	88.75 ± 2.6^a^	80 ± 1.02^ab^	80 ± 1.44^ab^	76.88 ± 4.72^ab^	78.75 ± 1.61^ab^	72.5 ± 1.02^b^	73.13 ± 1.2^b^
C: N	22.49 ± 0.58^a^	22.86 ± 0.95^a^	23.47 ± 0.57^a^	22.73 ± 0.47^a^	22.76 ± 0.37^a^	22.23 ± 1.06^a^	21.05 ± 1.02^a^	23.36 ± 0.58^a^	21.62 ± 0.46^a^

Mean (n = 4) values ± SE are shown. AM+ represents the treatment containing arbuscular mycorrhizal fungi and AM− represents the mycorrhizal free treatment. Different letters indicate significant differences (*p* < 0.05). Abbreviation: TC (soil total carbon), TN (soil total nitrogen), TP (soil total phosphate), TK (soil total potassium), AN (soil available nitrogen), AP (soil available phosphate), AK (soil available potassium), C: N (the ratio of total soil carbon to soil total nitrogen).

**Table 2:** Soil microbial community diversity indices for the different treatments (AM+ and AM−), at different sampling times (T_0_, T_90_, T_120_, T_150_ and T_180_).

Index	T_0_	T_90_		T_120_		T_150_		T_180_	
		AM+	AM−	AM+	AM−	AM+	AM−	AM+	AM−
H′	2.96 ± 0.018^a^	2.939 ± 0.007^a^	2.918 ± 0.032^a^	2.964 ± 0.008^a^	2.747 ± 0.067^b^	2.752 ± 0.056^b^	2.924 ± 0.011^a^	2.932 ± 0.028^a^	2.942 ± 0.013^a^
D	0.927 ± 0.002^a^	0.925 ± 0.001^a^	0.924 ± 0.002^a^	0.926 ± 0.001^a^	0.905 ± 0.007^b^	0.906 ± 0.006^b^	0.922 ± 0^a^	0.925 ± 0.003^a^	0.923 ± 0.001^a^
J	0.815 ± 0.011^a^	0.816 ± 0.01^a^	0.817 ± 0.006^a^	0.808 ± 0.005^a^	0.801 ± 0.013^a^	0.812 ± 0.007^a^	0.807 ± 0.007^a^	0.81 ± 0.014^a^	0.808 ± 0.004^a^

Mean (n = 4) values ± SE are shown. AM+ represents the treatment containing arbuscular mycorrhizal fungi and AM− represents the mycorrhizal free treatment. Different letters indicate significant differences (*p* < 0.05). Abbreviation: H’ (Shannon-Weaver Index), D (Simpson’s diversity index), J (Pielou evenness index).
