# Corrigendum: Arbuscular mycorrhiza enhance the rate of litter decomposition while inhibiting soil microbial community development

**DOI:** 10.1038/srep45947

**Published:** 2017-04-28

**Authors:** Heng Gui, Kevin Hyde, Jianchu Xu, Peter Mortimer

Scientific Reports 7: Article number: 4218410.1038/srep42184; published online: 02 08 2017; updated: 04 28 2017

This Article contains errors in Reference 7, which was incorrectly given as:

‘Hashe, A., Allah, E. F. A., Alqarawi, A. A., Aldubise, A. & Egamberdieva, D. Arbuscular mycorrhizal fungi enhances salinity tolerance of Panicum turgidum Forssk by altering photosynthetic and antioxidant pathways. *Journal of Plant Interactions*, 1–35 (2015)’.

The correct reference is listed below as ref 1.
