# Supplementary material for: Arbuscular mycorrhiza enhance the rate of litter decomposition while inhibiting soil microbial community development
Source: Sci Rep. 2017 Feb 8;7:42184. doi: 10.1038/srep42184 (PMC5296878; doi:10.1038/srep42184)
Supplement: Supplementary Information [file srep42184-s1.pdf]

Supplementary Information

Title: Arbuscular mycorrhiza enhance the rate of litter decomposition while inhibiting soil microbial community development

Heng Gui [1, 2, 3, 4], Kevin Hyde [1, 3, 4], Jianchu Xu [1,2], Peter Mortimer [1, 2, *]

1 Key laboratory for Plant Diversity and Biogeography of East Asia, Kunming Institute of Botany, Chinese Academy of Sciences, Kunming 650201, China
2 World Agroforestry Centre, East and Central Asia, Kunming 650201, China
3 Centre of Excellence in Fungal Research, Mae Fah Luang University, Chiang Rai 57100, Thailand
4 School of Science, Mae Fah Luang University, Chiang Rai 57100, Thailand

*Corresponding author: P.Mortimer@cgiar.org

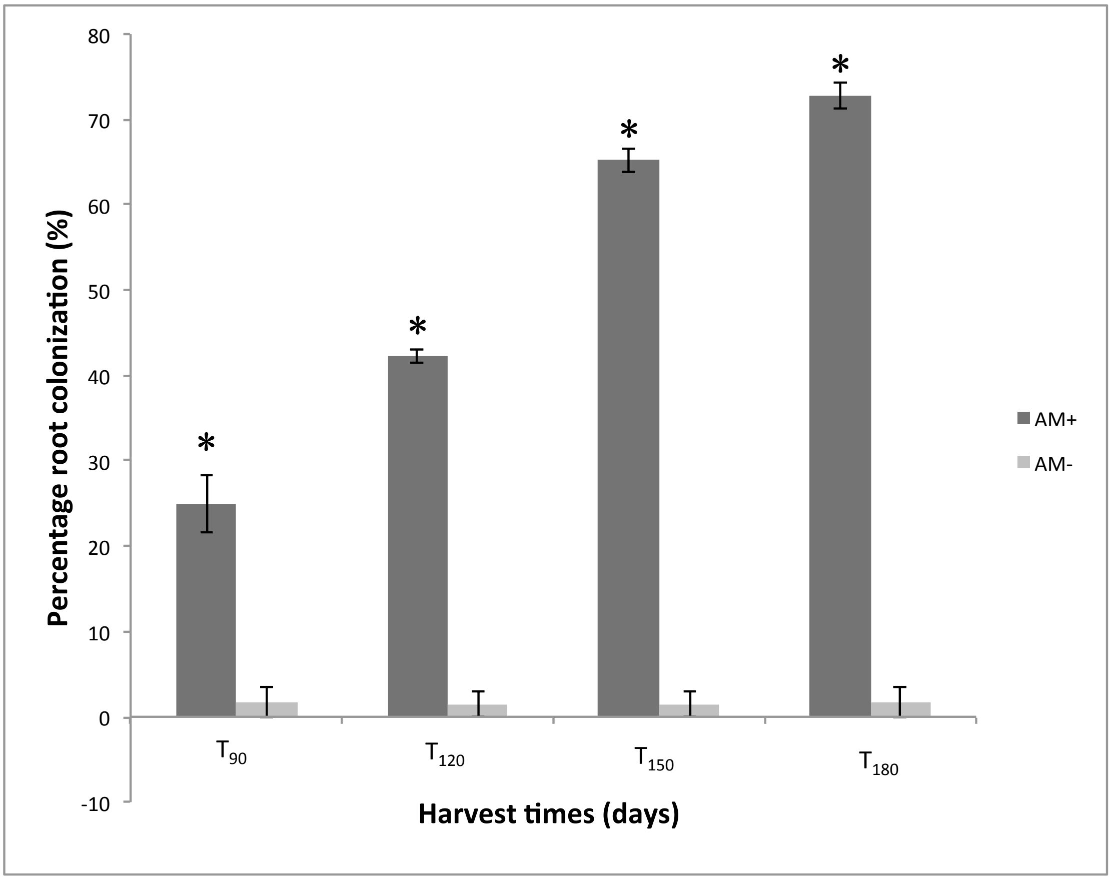

Supplementary Figure S1. Percentage colonization of roots by arbuscular mycorrhizal fungi at different sampling times (AM+ represents for AM fungal inoculation and AM- represents for non-inoculation, n=4, ±SE, p<0.05)

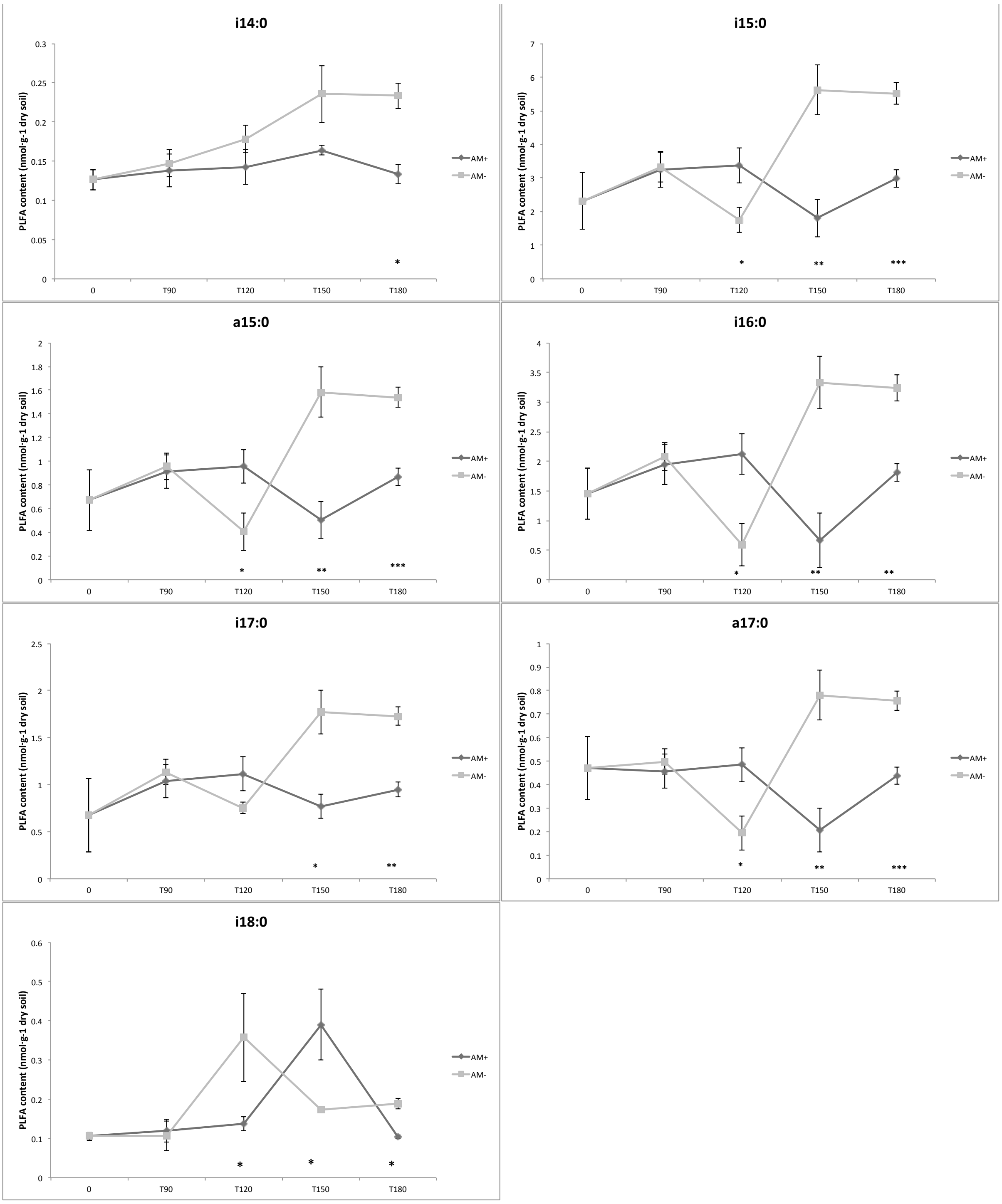

Supplementary Figure S2. The change in soil phospholipid fatty acid analysis (PLFA) contents (nmol • g-1 dry soil) of specific makers for Gram positive bacteria (i14:0, i15:0, a15:0, i16:0, i17:0, a17:0 and i18:0) for different sampling times (0, T_{90}, T_{120}, T_{150} and T_{180}). AM+ represents the treatment containing arbuscular mycorrhizal fungi and AM- represents the mycorrhizal free treatment. Mean (n=4) values ±SE are shown; in each plot * indicates p<0.05, ** indicates p<0.01, *** indicates p<0.001.

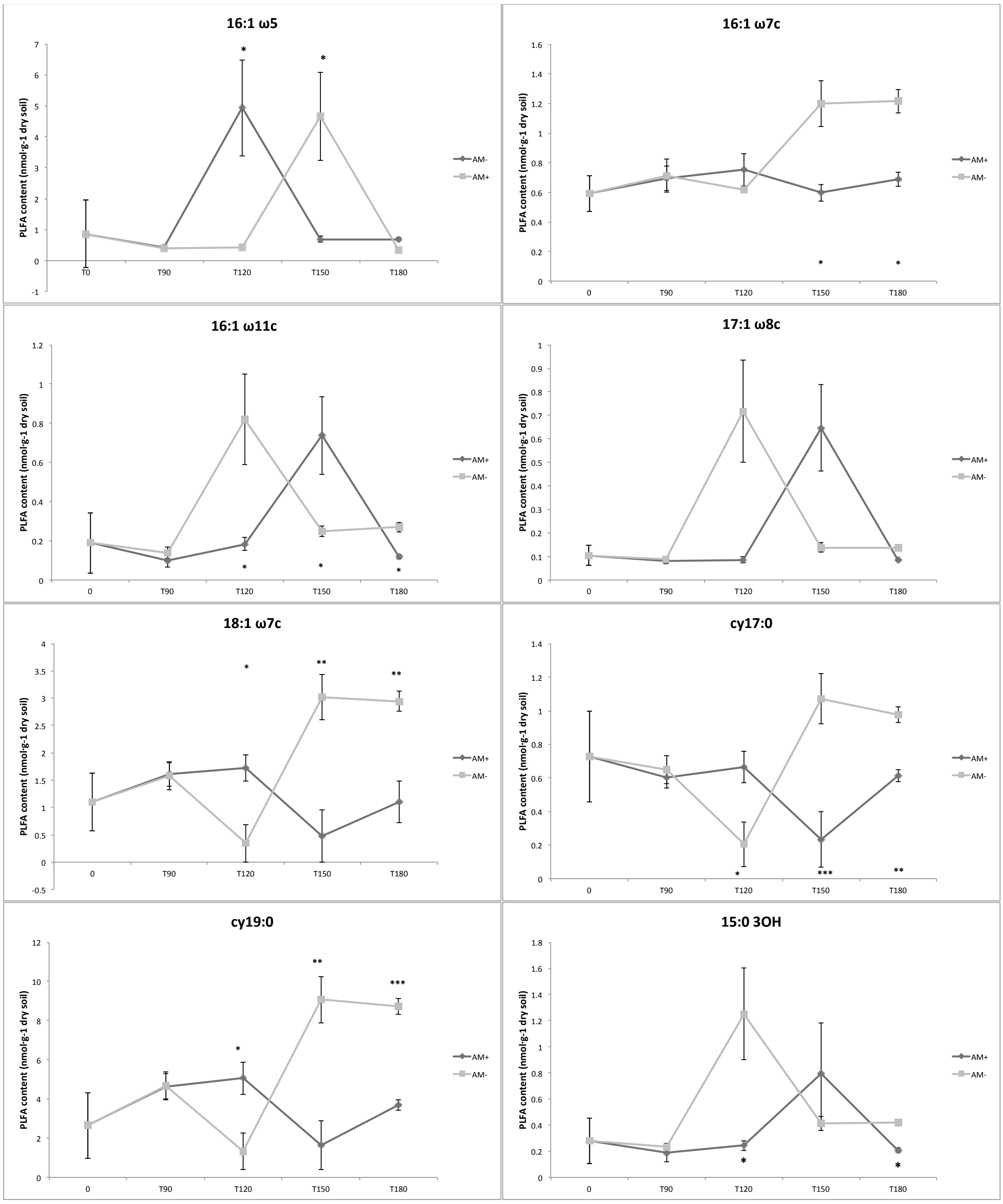

Supplementary Figure S3. The change in soil phospholipid fatty acid analysis (PLFA) contents (nmol • g-1 dry soil) of specific makers for Gram negative bacteria (16:1 ω 5, 16:1 ω 7c, 16:1 ω 11c, 17:1 ω 8c, 18:1 ω 7c, cy17:0, cy19:0 and 15:0 3OH) for different sampling times (0, T_{90}, T_{120}, T_{150} and T_{180}). AM+ represents the treatment containing arbuscular mycorrhizal fungi and AM- represents the mycorrhizal free treatment. Mean (n=4) values ±SE are shown; in each plot * indicates $p<0.05$, ** indicates $p<0.01$, *** indicates $p<0.001$.

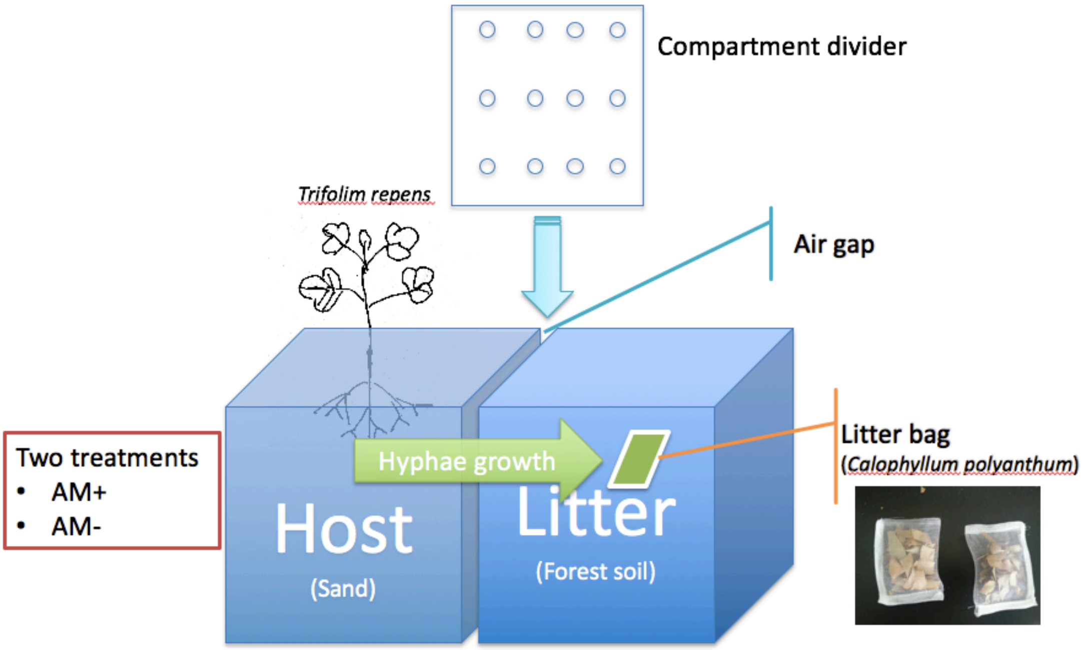

Supplementary Figure S4. Schematic representation of the air-gap microcosm, indicating the host plant chamber (Host), chamber with forest soil and litter bags (Litter), and the air gap between the two chambers. The compartment divider is made of an acrylic plate drilled with evenly spaced holes (4mm in diameter), and then covered with 20µm nylon mesh on both side to form the air gap. AM+ represents the AM fungal inoculation treatment and AM- represents for non-AM treatment.
